# Practitioner's Bookshelf

**Published:** 1892-01-23

**Authors:** 


### PRACTITIONERS' BOOKSHELF.

### INHERITED CONSUMPTION.

Of the large number of children who die of some form of consumption, about half are found to have some other members of their family similarly affected. It is found, too, that the richer classes do not suffer so much as the poorer ones; the difference lying in the fact that the latter suffer more from impure air and improper food than the former. If then the mortality from inherited consumption can be lowered by a plentiful supply of fresh air and good food, it behoves us to use these means to the best advantage. Dr. Dale has recently published a little book dealing with this subject. Popular superstitions are very difficult to overcome, and people look with horror on the doctor who insists on a child's bedroom window being opened a little at night. Why a child should breathe bad air at night and not in the day, never seems to occur to them. If only expired air could be coloured, people would be able to see the vitiation of the atmosphere which takes place, and would more easily be persuaded to allow it to be changed. The habitual rebreathing of a vitiated atmosphere, more than anything makes the body suitable soil for the growth and development of the tubercle bacillus. Dr. Dale writes, "The truth about bad air cannot be too frequently brought before the public, and proclaimed on the house-tops." It might be added, and taught in the Board schools. Nor is the danger of vitiated air at night only. In how few rooms do we see any satisfactory arrangement for changing the air? In how few rooms do we find a sufficient cubic space for the number of persons occupying it? Most people when indoors, especially in winter, breathe air which has been vitiated by themselves or others. What wonder, then, that consumption claims such a large number of them. According to Dr. Dale, this has been preached for at least over a hundred years; but we have not much mended our ways, and consequently consumption carries off its victims as before.

Scanty food, and especially improper food, is nearly as potent a cause as vitiated air. The common custom of allowing children to leave the fat of their meat is a great factor in bringing out the inherited taint. For the children of consumptive parents fat in some form or other is essential, if the enemy is to be kept off. As Dr. Fothergill wrote: "Fat is one of the greatest of our curative agents; it is especially potent as a preventative agent." Nor is it necessary to begin with cod liver oil, or even to use it at all. Fat can be given in many other and less objectionable forms, as, for instance, butter, cream, milk. Extra butter may be worked in in many ways. Dr. Fothergill recommended biscuits and butter, and a lump of butter to be melted in each plateful of milk pudding. Dripping is a cheap and useful form of fat. Sardines and other fish preserved in oil form pleasant vehicles for the ingestion of fat. By some such device as this, fat may be given until the child's objection to it has been overcome. Excess of food, however, is nearly, or quite, as bad as improper food. What is excess, and what is not, must be left to a doctor to determine. A most important point to attend to is regularity. Let them have definite meals at definite times, without any intermediate stuffing with sweets or cakes.

Another much-neglected and very important cause of lowered nutrition is a bad state of the teeth. Parents seldom pay any attention to the state of their children's teeth until the front ones get affected and their digestions have become permanently weakened.

The question of education should be altogether secondary. The less they are kept indoors in fine weather the better. Long hours of play and short hours of work should form the rule; all attempts at forcing the intellectual powers should be avoided, and all endeavours should be directed to rearing a healthy body. It is of no use to highly educate a child if it is to die at an early age, and it does not matter if a child is a little backward at first, if it gets as compensation a healthy body to take it through life.

Children with an inherited tendency to consumption require to be properly clothed, for cold, as any other agent which lowers the vital powers, aids the growth of tubercle. The fashion of allowing children to go barelegged is most pernicious. All children, and especially those of the class under consideration, are easily chilled, and should, from their earliest age, wear flannels always next their skin. If this be begun early, the child will become used to it and never object. The flannel should reach from the neck to the wrists and ankles. The popular practice of cutting it low at the neck is bad, as the apices of the lungs—the very parts most prone to disease—are thus left uncovered. It should be known that apices of the lungs extend above the collar-bone for an inch and a-half, so that the flannel should reach at least as high as that.

Any organ which is overworked may become the seat of tubercular disease; and this applies to the lungs as to the joints. Violent exercise is therefore to be avoided. Boys of consumptive families should avoid athletic competitions, and football, with its great exertions and risks of injury, should not be thought of. Exercise must be taken, not only for its influence on the general nutrition, but because it moderately inflates the lungs, and so aids their development. So important is this inflation of the lungs that a book has been written on "The Physiological Treatment of Consumption," in which various methods of inflating the lungs form the basis of treatment.

In spite, however, of the most careful rearing, the inherited tendency is hardly ever quite got rid of. Any great lowering of the vital powers may, at any period of the patient's life, be followed by a rapid development of the disease. In many cases, however, the disease may be kept at bay until late in life. According to recent researches on the Continent, we now know that infection plays a much more important part in the spread of consumption than was formerly suspected. Every consumptive patient is a prolific source of infection, spreading the cause of the disease wherever he goes. This cause, the tubercle bacillus, only requires suitable soil, that is susceptible people, for its growth. Of all susceptible people the children of consumptive parents are the most prone to take the disease, and should consequently be kept away from any consumptive person, or from using or sleeping in the rooms used by such patients.

### HEALTH IN SCHOOLS.

In the internal economy of a large school there is no more important subject than that of health. That the physical surroundings of the pupils should be of such a kind as to promote their bodily health is an essential condition in the attainment of that union of bodily with mental health so necessary to school life. That these matters have been grievously neglected in the past is only too painfully apparent from the serious outbreaks that from time to time occur in our great public schools.

The Medical Officers of Schools Association is a society formed for the purpose of disseminating knowledge of the laws of health in their special adaptation to schools; and the third edition of their "Rules for the Prevention of Infectious and Contagious Diseases in Schools" has just reached us. The rules are divided into five sections, with two appendices. The first section deals with general hygiene, and begins with some general rules for the construction of drainage. These rules are excellent as far as they go, but they might with advantage be carried a good deal further. It would be well within the scope of the Association to draw up a code of by-laws which, *mutatis mutandis*, might be applicable to all schools, whether in town or country. Such a code, if carefully drawn up and illustrated by a few well-chosen diagrams, would be invaluable to head-masters and medical officers.

Other points dealt with in this section are the necessity for a medical officer with undivided responsibility for the whole school, water supply, milk, and laundry. There can be no question as to the necessity for an undivided responsibility for the medical care of a school. The medical officer should be charged not only with the treatment of sickness, but also with its prevention. It should be his duty to inspect periodically all the school buildings and make what recommendations he thinks necessary for their improvement. He should, in fact, be the medical officer of health to the community.

The questions of milk supply and of the school washing are also of great importance. If it is not practicable for a school to possess its own dairy, the source or sources from which the milk supply is obtained should be under the direct supervision of the medical officer, who should periodically inspect both buildings and cows; and no boarding house should be supplied with milk except from a dairy approved and certified by him.

So too with regard to the washing. Every school should, if possible, have its own laundry; but if this is not practicable, then the most careful supervision should be exercised to prevent the possibility of infection being conveyed from outside sources to the school linen.

Section II. deals with the infirmary. The necessity for efficient means of isolation for cases of infectious disease is pointed out, and the general principles of arrangement are indicated. For more detailed information on this head, reference is made to the two pamphlets on "The Construction and Maintenance of School Infirmaries," and "The Arrangement and Construction of Sanatoria," issued by the Association. It cannot be too strongly impressed upon the governing bodies of schools that the prompt isolation of the first case of an infectious disease is often the means of preventing an epidemic. Some provision for an epidemic must undoubtedly be made, especially if the sanatorium is to be arranged with a view to take cases of measles. Such provision may conveniently take the form of one, or preferably, two wards, the size of which must depend upon the size of the school. But in addition to these, there should always be at least two small rooms well isolated from the large wards, and from each other, in which cases either of doubtful nature or of well marked specific diseases can be treated. The tendency, both of governing bodies and of masters, is to whittle down the accommodation of the sanatorium to an irreducible minimum; to grudge, in fact, any expenditure on a building which may, and often does, stand empty for months together, and which is a source of expense rather than of revenue. Such policy is short-sighted from every possible standpoint. From the governor's point of view because the absence of efficient means of isolation may often mean the closure of a school on account of an epidemic, and from the master's point of view because the sanatorium is a means of defence against the spread of disease within their own houses. Another class of persons most directly interested in the provision of a proper sanatorium is that of the parents, to whom the absence of proper means of preventing epidemics means the possibility of an infected boy being returned upon their hands to become a focus of infection. Section III. deals with the medical examination of scholars on admission, and the quarantine to be enforced when a pupil has been exposed to infection. Section IV. prescribes the means to be adopted against the introduction of infectious disease to a school from without; and Section V. deals with the measures to be adopted when an infectious disease has made its appearance in a school.

Many of the points dealt with in the last three sections are of a purely medical nature. But all are characterised by a directness and thoroughness which is beyond praise. One most excellent recommendation is to the effect that "In replying to enquiries by parents in respect of any case of serious illness in a school, the precise terms used by the medical officer in describing the nature or progress of the malady should be communicated either directly by him or through the house master."

In appendix A several forms of health certificates are set forth.

Appendix B contains some suggestions as to means of disinfection. The usual disinfectants, such as carbolic acid, Condy's fluid, sulphur, and others are described; and a qualifying note is added that recent experiments seem to show that none of them are to be relied upon as absolutely effectual in certain cases. The most effectual of all disinfectants, however, steam either at or above atmospheric pressure is only slightly alluded to. The whole question of disinfection is an extremely difficult one, and it is very questionable whether, unless a sanatorium be provided with a properly equipped laundry and an efficient steam apparatus for disinfection, it would not be safer to destroy all infected clothing rather than run the risk of further spreading infection.

